# Underwater endoscopic mucosal resection of upper gastrointestinal subepithelial tumors: A case series pilot study (with video)

**DOI:** 10.1097/MD.0000000000031072

**Published:** 2022-10-14

**Authors:** Su Jin Kim, Tae Un Kim, Cheol Woong Choi, Hyung Wook Kim, Su Bum Park, Dae Gon Ryu

**Affiliations:** a Department of Internal Medicine, Pusan National University School of Medicine and Research Institute for Convergence of Biomedical Science and Technology, Pusan National University Yangsan Hospital, Yangsan, Korea; b Department of Radiology, Pusan National University School of Medicine and Research Institute for Convergence of Biomedical Science and Technology, Pusan National University Yangsan Hospital, Yangsan, Korea.

**Keywords:** case report, endoscopic resection, subepithelial tumor

## Abstract

Underwater endoscopic mucosal resection (UW-EMR) has been recently introduced as an effective technique for rectal third layer subepithelial tumors. Therefore, we aimed to assess the safety, efficacy, and procedure time of UW-EMR for upper gastrointestinal subepithelial tumors (SETs) originating from the deep mucosal and/or submucosal layers.

**Methods:**

Between August 2018 to July 2022, a total of 17 SETs (7 duodenal SETs, 6 gastric SETs, and 4 esophageal SETs) were included in this study. On endoscopic ultrasound examinations, the tumors were found to be embedded in the submucosa without muscularis propria invasion. All SETs were resected successfully using UW-EMR. The characteristics of the tumors and their R0 resection rate, adverse event rate, and recurrence rate were evaluated retrospectively.

**Results:**

The mean tumor size was 0.9 cm (range, 0.3-1.5 cm). En bloc resection and complete resection rates were 100%, respectively. The patients showed no complications such as perforation or bleeding. Histologic assessments of the resected tumors revealed 9 neuroendocrine tumors (7 on the duodenum, 2 on the stomach), 2 gastric cystica profunda, 1 gastric follicular lymphoma, 1 gastric fibromyxoma, 3 esophageal granular cell tumors, and 1 esophageal adenoid cystic carcinoma. The mean procedural time was 3.2 min (range, 1.3-8.7 minutes). The overall en bloc and complete resection rates were 100%, respectively. No recurrence was observed during the follow-up period.

**Conclusion:**

UW-EMR is a safe and effective treatment for upper gastrointestinal SETs embedded in the submucosal layer. Further studies are needed to compare other endoscopic resection techniques.

## 1. Introduction

The incidence of asymptomatic incidental upper gastrointestinal subepithelial tumors (SETs) is increasing with the popularization of endoscopic screening for upper gastrointestinal tract tumors and advancements in high-resolution endoscopy.^[1]^ In general, most small SETs covered with normal mucosa can be observed with periodic follow-up. However, some tumors such as neuroendocrine tumors (NETs), lymphoma, and granular cell tumor (GCTs) have malignant potential. Thus, differential diagnosis between potentially malignant and benign upper gastrointestinal SETs is important.

Endoscopic ultrasonography (EUS) is a useful tool to approximate the size, layer of origin, and echogenicity of tumors.^[2]^ NETs and GCTs originating from the submucosal layer of the upper gastrointestinal wall have malignant potential. Unfortunately, the accuracy of EUS to predict the correct histologic diagnosis ranges from 45% to 66%, and is also dependent on the operator’s experience.^[3–5]^ Therefore, definite tissue diagnosis of hypoechoic SETs embedded in the submucosa should be considered whenever possible.

Conventional endoscopic mucosal resection (EMR) is a simple procedure for the diagnosis and treatment of small SETs confined to the muscularis mucosa and/or submucosa. Submucosal injection before snaring is helpful to avoid perforation by increasing the distance between the muscle and the tumor.^[6]^ However, submucosal injection makes lesion capture by snaring more difficult.^[7]^ Furthermore, complete tumor resection is not always easy because SETs involve the submucosa.^[8]^ Endoscopic submucosal dissection (ESD) can increase the rate of en bloc R0 resection for these lesions. However, the disadvantages of ESD include the technical burden, prolonged procedure time, and related adverse events.^[9]^

Underwater EMR (UW-EMR) was recently introduced as a useful method to remove rectal NETs with similar R0 resection rate as ESD.^[10]^ The floating force provided by filling the gastrointestinal lumen with water instead of submucosal injection allows lifting of mucosal and submucosal tumors from the muscularis propria and facilitates snaring of the tumor by the creation of a pseudopedicle.^[11]^ Moreover, UW-EMR for rectal NETs can reduce the procedure time dramatically in comparison with ESD. Therefore, we aimed to evaluate the efficacy, safety, and procedure time of UW-EMR for deep mucosal and/or submucosal layer SETs located on the esophagus, stomach, and duodenum.

## 2. Methods

### 2.1. Study design

From August 2018 to July 2022, a total of 17 upper gastrointestinal SETs were removed using UW-EMR at Pusan National University Yangsan Hospital. All patients were examined by endoscopy and EUS (UMP, 20 MHz; Olympus, Tokyo, Japan) before the endoscopic resection. EUS confirmed that the SETs had hypoechoic echogenicity involving the submucosa without muscularis propria invasion. The location, size, pathology, complete resection rate, complication rate, recurrence rate, and follow-up duration were evaluated retrospectively. The protocol for this study was reviewed and approved by the Institutional Review Board of School of Medicine Pusan National University (05-2020-186). Written informed consent was obtained from all the patients. This study was conducted in accordance with the human and ethical principles of research specified by the Declaration of Helsinki.

Endoscopic procedure (see Video, Supplemental Video, http://links.lww.com/MD/H584, which illustrates UW-EMR for esophageal, duodenal and gastric SETs)

**Figure 1.**
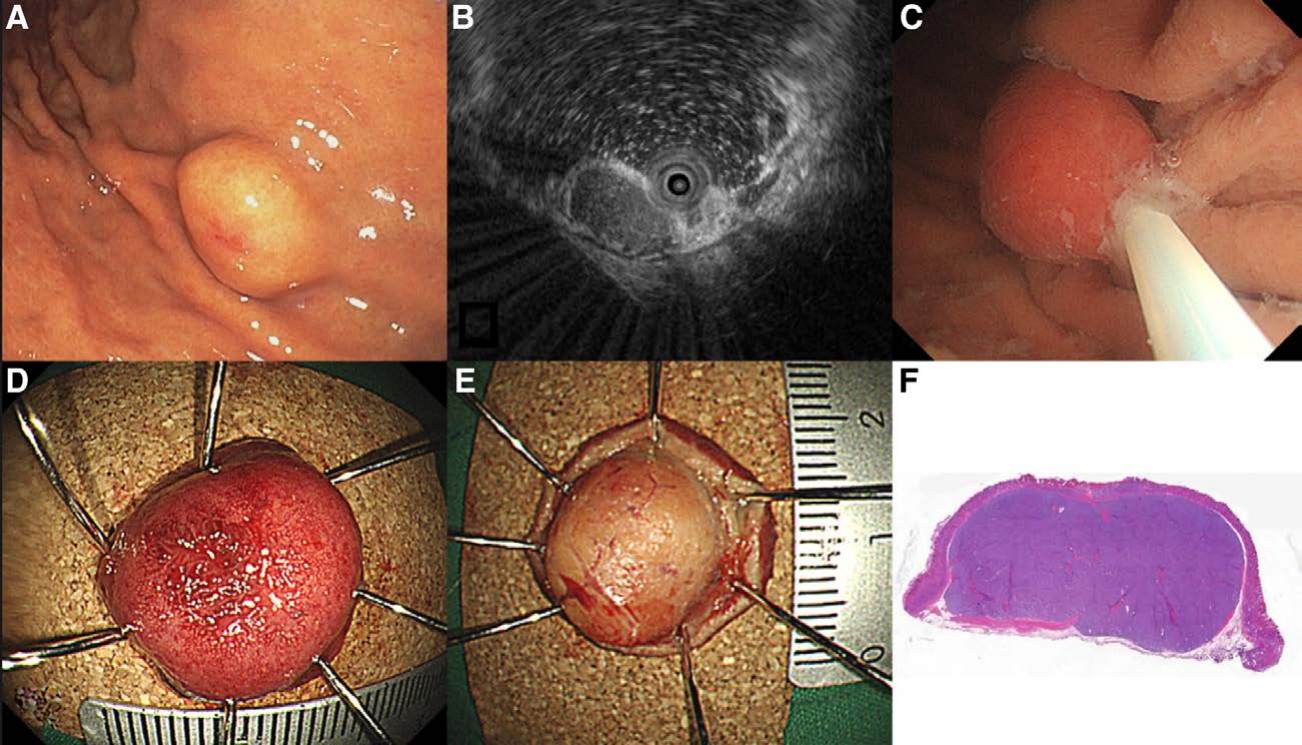
Endoscopic image shows a 13 mm sized yellowish, hard subepithelial tumor with an erosion on the top in the stomach body (A). A endoscopic ultrasonography shows a 13 mm, homogeneous, hypoechoic lesion in the third layer (B). Water filling in the lumen causes the lesion to float, allowing the endoscopist to snare the tumor easily (C). En bloc resection was achieved (D and E). A pathological examination shows that a G1 neuroendocrine tumor (mitotic rate: 0/10 high-power field, Ki67 proliferation index: 1%) with free lateral resection margin (F).

The endoscope used for UW-EMR was GIF-HQ290 or GIF-2TQ260M (Olympus, Tokyo, Japan) in this study (Fig. 1A). All endoscopic procedures were performed under conscious sedation (intravenous administration of midazolam and meperidine). The patient maintained the left lateral decubitus position during the procedure. EUS was performed to evaluate the origin of SET before the endoscopic resection (Fig. 1B). When the SET was located on the esophagus, the table was tilted about 15 degrees to prevent aspiration. Warm distilled water was infused into the gastrointestinal lumen using a water pump. The infusion of water continued until the tumor was completely immersed underwater. The polypectomy snare (Endoflex, Germany) size (range, 15~25 mm) was determined based on the size of the SETs. A polypectomy snare was inserted through an endoscopic accessary channel. SET capture was performed in the underwater immersion state after confirming that the target tumor was slightly bulging from the mucosal surface (Fig. 1C). SET resection was performed using an Endocut Q current (effect, 3; cut duration, 2; cut interval, 3), which was generated using a VIO300D (ERBE, Tuebingen, Germany) electorosurgical unit (Fig. 1D and E). Endoscopic clipping was used to prevent complications such as delayed bleeding or perforation after UW-EMR for duodenal tumor. In other location, such as esophagus and stomach, prophylactic endoscopic clipping was not used. Resected specimens were evaluated by pathologist (Fig. 1F). All procedures were conducted by 3 endoscopists (SJ Kim, CW Choi and DG Ryu) with > 5 years of experience in therapeutic endoscopy. Patients with gastrointestinal NETs, esophageal GCTs, esophageal adenoid cystic carcinoma, gastric follicular lymphoma, underwent a follow-up endoscopic examination to evaluate local recurrence at the resection site. The first follow-up endoscopic examination with biopsy was performed approximately 3 months after the UW-EMR.

### 2.2. Definition

Histopathological evaluation of the specimens was performed with 2-mm slices, with the microscopic evaluation including depth of invasion, lateral and vertical resection margins, and pathologic diagnosis. En bloc resection was defined as endoscopic resection of the tumor in a single piece. Complete resection was defined by the absence of tumor cells in microscopic evaluations at the resection margin. The procedure time was counted from the infusion of warm water to the resection of SET. We defined significant bleeding as a reduction of more than 2 g/dL in the hemoglobin level. The patient underwent second-look endoscopy when significant bleeding occurred after endoscopic resection. Perforation was diagnosed by the presence of subdiaphragmatic air or subcutaneous emphysema on chest radiographs after UW-EMR.

## 3. Results

The average age of the patients included in the study was 60.1 years (range, 36-79 years). The mean tumor size was 0.9 cm (range, 0.3-1.5 cm). Seven of the 17 lesions were located in the duodenum (46.6%), 6 were located in the stomach (26.7%), and 4 were present in the esophagus (26.7%) (Table 1). Endoscopic biopsies before UW-EMR diagnosed 9 of the 15 SETs (6 duodenal NETs, 2 gastric NETs, and 1 esophageal GCT).

**Table 1:** Patient and tumor characteristics.

Patient	Sex	Age, yrs	Location	Paris classification	Tumor size, cm
1	Male	61	Bulb	0-Isp	0.6 × 0.4
2	Male	36	Bulb	0-IIa	0.3 × 0.2
3	Female	67	Bulb	0-IIa	0.4 × 0.3
4	Male	46	Bulb	0-Is	0.8 × 0.6
5	Male	67	Mid esophagus	0-Is	1.2 × 1.0
6	Female	70	Mid esophagus	0-Is	0.6 × 0.3
7	Male	65	Stomach body great curvature	0-Is	1.1 × 0.6
8	Female	62	Stomach antrum great curvature	0-Is	0.7 × 0.6
9	Female	73	Stomach body great curvature	0-Is	1.4 × 1.2
10	Female	60	Superior descending angle	0-Is	0.8 × 0.6
11	Male	64	Bulb	0-IIb	0.4 × 0.2
12	Female	54	Mid esophagus	0-Is	1.4 × 1.2
13	Female	61	Mid esophagus	0-IIa	0.7 × 0.7
14	Female	44	Stomach body great curvature	0-Is	0.9 × 0.8
15	Female	44	Near ampulla	0-Isp	1.5 × 1.2
16	Female	79	Stomach body great curvature	0-Is	1.2 × 1.1
17	Female	68	Stomach body great curvature	0-Is	0.5 × 0.5

The overall en bloc and complete resection rates were 100%, respectively. The mean procedural time was 3.2 min (range, 1.3-8.7 minutes). Histologic assessments of the removed SETs revealed 9 NETs (7 in the duodenum and 2 in the stomach), 2 gastric cystica profunda, 1 gastric follicular lymphoma, 1 gastric fibromyxoma, 3 esophageal granular cell tumors, and 1 esophageal adenoid cystic carcinoma. All NETs were G1 grade and show no lymphovascular invasion. Follow-up endoscopic examinations were performed 3 months after UW-EMR. The biopsies at the UW-EMR site showed no residual tumor. No serious adverse events such as perforation or significant bleeding occurred during the procedures (Table 2).

**Table 2:** Clinical outcomes.

Patient	Procedure time	En bloc resection	Resection margin	R0 resectionn	Complication	Histology
1	8’40“	Yes	Negative	Yes	No	NET (G1)
2	4’10“	Yes	Negative	Yes	No	NET (G1)
3	2’00“	Yes	Negative	Yes	No	NET (G1)
4	1’40“	Yes	Negative	Yes	No	NET (G1)
5	2’10“	Yes	Negative	Yes	No	GCT
6	3’20“	Yes	Negative	Yes	No	GCT
7	2’40“	Yes	Negative	Yes	No	NET (G1)
8	1’40“	Yes	Negative	Yes	No	Fibromyxoma
9	4’00“	Yes	Negative	Yes	No	Follicular lymphoma
10	6’00“	Yes	Negative	Yes	No	NET (G1)
11	2’50“	Yes	Negative	Yes	No	NET (G1)
12	5’00“	Yes	Negative	Yes	No	Adenoid cystic carcinoma
13	1’20“	Yes	Negative	Yes	No	GCT
14	2’20“	Yes	Negative	Yes	No	GCP
15	2’00“	Yes	Negative	Yes	No	NET (G1)
16	2’30“	Yes	Negative	Yes	No	GCP
17	2’00“	Yes	Negative	Yes	No	NET (G1)

G = grade, GCP = gastritis cystic profunda, GCT = granular cell tumor, NET = neuroendocrine tumor.

## 4. Discussion

The widespread use of endoscopy for health checkups and the advancements in high-definition endoscopy have increased the rate of detection of upper gastrointestinal SETs. Although EUS can provide information regarding SETs, including their echogenicity, size, and layer of origin, SETs originating from the submucosal layer usually require pathologic diagnosis, except lipomas, cysts, and vascular lesions.^[12]^ The results of the present study suggested that UW-EMR was a safe and effective resection method for upper gastrointestinal SETs originating from the submucosal layer.

Although conventional EMR is a simple endoscopic resection technique for upper gastrointestinal SETs embedded in the submucosal layer, it is not easy to obtain a deep resection margin in this procedure.^[13]^ Moreover, the extensive damage at the tumor resection margin makes it difficult to determine the pathologic margin status. In comparison with conventional EMR, ESD shows a higher en bloc resection rate while securing the resection margin.^[14,15]^ In fact, ESD can achieve en bloc resection even in cases where EMR is difficult due to submucosal fibrosis. However, ESD requires greater technical skill and a prolonged procedure time, and is associated with a higher risk of adverse events, including perforation.

Considering these limitations of conventional EMR and ESD, several modified EMR methods have been described as effective treatment modalities. For tumors less than 1 cm in size that are located in the third layer of the esophagus or duodenum, a band-ligation–assisted EMR showed high en bloc and complete resection rates.^[16,17]^ EMR with a cap (EMR-C) has also been shown to be an effective method to remove submucosal SETs less than 1 cm in size that are located on the digestive tract.^[18]^ However, the size of the transparent cap or band limits the usability of this technique in removing SETs sized less than 1 cm.

UW-EMR has recently emerged as a resection method to secure the margin for non-ampullary duodenal epithelial tumors less than 2 cm in size.^[19,20]^ The water filling during UW-EMR maintains the proper muscle layer and allows tumors to float in the water without increasing tissue tension. The contraction of the superficial layers submerged in water and the lifting movement caused by the fat tissue of the submucosa create a pseudopedicle, making it easier to capture the tumor. Furthermore, lesions greater than 1 cm in size can be captured using a large-diameter snare. Therefore, complete resection was achieved in all 3 cases larger than 1 cm in this study.

UW-EMR offers advantages over various endoscopic resection methods, including ESD, conventional EMR, band-ligation–assisted EMR, and EMR with a cap. The submucosal injection performed in several endoscopic resection methods causes tumors to sink under the epithelium. The increased tension of the surrounding tissue after submucosal injection makes capture of the tumor more difficult. In addition, lumen distention caused by air insufflation during the procedure causes thinning of the muscle layer. These factors increase the procedure time and the risk of perforation. In particular, the duodenum and esophagus are more difficult sites to perform endoscopic resection because of the narrow lumen and thin proper muscle layer. However, filling saline into the lumen causes the duodenal or esophageal wall to gently slope without thinning the muscle layer and sinking the gastrointestinal SETs. This increases the safety of the procedure while decreasing the risk of adverse events such as perforation.

A recent study reported that UW-EMR can effectively remove colorectal lesions or duodenal lesions with submucosal fibrosis.^[21,22]^ Mechanical stimulation caused by procedures such as biopsy can cause submucosal fibrosis, resulting in non-lifting of SETs and complicating various EMR techniques after submucosal injection. Unfortunately, we were unable to assess the effectiveness of UW-EMR for SETs with fibrosis because there was no case of fibrosis in our study.

This study had some limitations. First, this was a retrospective study, and the retrospective review of the clinical outcomes may have introduced a potential bias. Second, this was a single-center study with a small sample size, and all procedures were performed by 3 experienced endoscopists, which limited the generalizability of these findings to other centers with less experience. A multiple-center study including a large sample size is required to overcome these limitations.

In conclusion, the results of our study suggest that UW-EMR is safe and effective for the resection of upper gastrointestinal SETs located in the submucosal layer, including NETs, and GCTs.

## Authors’ contributions

All authors contributed to the study conception and design. Material preparation, data collection and analysis were performed by Cheol Woong Choi, Hyung Wook Kim, Su Bum Park, Dae Gon Ryu. Tae Un Kim wrote the first draft of the manuscript. Su Jin Kim reviewed edited this manuscript. All authors read and approved the final manuscript.

**Conceptualization:** Su Jin Kim, Tae Un Kim, Cheol Woong Choi, Hyung Wook Kim, Su Bum Park, Dae Gon Ryu.

**Writing – original draft:** Tae Un Kim.

## Supplementary Material


